# Immunomodulation as Therapy for Fungal Infection: Are We Closer?

**DOI:** 10.3389/fmicb.2018.01612

**Published:** 2018-07-25

**Authors:** Qi Hui Sam, Wen Shan Yew, Chaminda J. Seneviratne, Matthew Wook Chang, Louis Yi Ann Chai

**Affiliations:** ^1^Division of Infectious Diseases, University Medicine Cluster – National University Health System, Singapore, Singapore; ^2^Department of Biochemistry, Yong Loo Lin School of Medicine, National University of Singapore, Singapore, Singapore; ^3^Synthetic Biology for Clinical and Technological Innovation (SynCTI), Life Sciences Institute, National University of Singapore, Singapore, Singapore; ^4^Oral Sciences, Faculty of Dentistry, National University of Singapore, Singapore, Singapore; ^5^Department of Medicine, Yong Loo Lin School of Medicine, National University of Singapore, Singapore, Singapore; ^6^National University Cancer Institute, Singapore, Singapore

**Keywords:** immunotherapy, mycosis, invasive fungal disease, aspergillosis, candidiasis, anti-fungal, microbiome, mycobiome

## Abstract

Invasive fungal disease (IFD) causes significant morbidity in immunocompromised patients due to their weakened immune system. Immunomodulatory therapy, in synergy with existing antifungal therapy, is an attractive option to enhance their immune system and aid clearance of these opportunistic pathogens. From a scientific and clinical perspective, we explore the immunotherapeutic options to augment standard antifungal drugs for patients with an IFD. We discuss the range of immunomodulatory therapies being considered in IFD – from cytokines, including G-CSF, GM-CSF, M-CSF, IFN-γ, and cytokine agonists, to cellular therapies, consisting of granulocyte transfusion, adoptive T-cell, CAR T-cell, natural killer cell therapies, and monoclonal antibodies. Adjunct pharmaceutical agents which augment the immunity are also being considered. Lastly, we explore the likelihood of the use of probiotics and manipulation of the microbiome/mycobiome to enhance IFD treatment outcomes.

## Introduction

For many people living with cancer, rheumatological diseases and, solid organ and stem cell transplant recipients, medical advances have allowed them to prolong their lives. However, many of the treatments involve the alteration or weakening of the patient’s immune system; this predisposes the immunosuppressed patient to infections by opportunistic pathogens. Of the opportunistic infections, one of the most problematic is fungal infections. The disease spectrum of IFDs encompasses candidiasis, aspergillosis, cryptococcosis, zygomycosis, penicilliosis, histoplasmosis, and infections by other fungi such as, *Scedoporium, Fusarium*, and *Sporothrix* species. IFDs cause significant morbidity and mortality among immunocompromised patients, ranging between 30% and more than 60% in susceptible patients (Neofytos et al., 2009). In a review of the burden of fungal disease in Europe, the overall mortality in patients with IFD averaged around 35 to 45%, and be as high as 82.4% (Drgona et al., 2014). Globally, various international centers have yielded similarly high mortality rates from fungal infections (Brown et al., 2012; Rajasingham et al., 2017). The persistence of these high mortality rates, despite antifungal treatment, is the consequence of the immune predisposition or compromised status of the host to respond adequately to infection. The limited options of antifungal therapeutics available highlight the need to develop novel therapeutic strategies to optimize patient outcomes.

The strategy required to fight against fungal infections can be likened to playing battles in an electronic game, for example, battling other pokemon in Pokémon Go. To win a battle where you face a strong opponent, you either strengthen yourself, weaken your opponent, or do both. Antifungals are our potions to weaken the invasive fungi. Like humans, fungi are eukaryotic, making the hunt for fungal-specific targets more complicated, with many antifungals causing significant adverse side effects to the patient. The other way to win is by augmenting the host immunity; to continue the analogy, immunotherapy is our potion to strengthen ourselves to clear the infection. For an immunocompromised patient with an IFD, immunotherapy becomes an attractive option to add the immunity back. Immunomodulatory agents either turn up the immune response in the case of immunosuppression, or turn it down to prevent further harm to the patient in the case of an overactive immune system.

In this manuscript, we will review immunotherapies currently in use in the context of fungal infections, as well as other potential immunomodulatory modalities at their various stages of development.

## Cytokine Therapy

### Granulocyte Colony-Stimulating Factor (G-CSF) and Granulocyte-Macrophage Colony-Stimulating Factor (GM-CSF)

Granulocyte colony-stimulating factor, generic names include filgrastim and lenograstim, primarily stimulates neutrophil production and maturation. Chemotherapy may be myelosuppressive, causing neutropenia, and G-CSF can be used adjunctly to restore neutrophil counts (reviewed in Wright et al., 2017).

Granulocyte-macrophage colony-stimulating factor, generic names include sargramostim and molgramostim, is an important immunomodulatory cytokine. There is a large amount of literature about its functions, as it stimulates a wider range of immune cells than G-CSF. In brief, GM-CSF stimulates maturation of dendritic cells from monocyte precursors, differentiation of macrophages, and proliferation and activation of macrophages, monocytes, neutrophils, eosinophils, dendritic cells, and microglia (reviewed in Hamilton, 2008; van de Veerdonk et al., 2012; Shiomi and Usui, 2015; Scriven et al., 2017). In GM-CSF deficient mice, bacterial and viral clearance by alveolar macrophages is impaired, suggesting its role in aiding pathogen clearance (LeVine et al., 1999; Paine et al., 2000; Berclaz et al., 2002).

Neither G-CSF nor GM-CSF are directly linked to improvement in IFD-related incidence and mortality, but they may aid the faster recovery of the patient. During chemotherapy-induced FN, G-CSF is commonly prescribed. G-CSF reduces the duration of neutropenia and the length of stay during FN episodes; however, its administration did not conclusively improve infection-related mortality in FN (Clark et al., 2005). The latest Cochrane Database Systematic Review on the use of colony-stimulating factor in FN (both G-CSF and GM-CSF), reflected similar findings when they compared the results of 14 RCTs (Mhaskar et al., 2014). While the paper concluded there was a shorter duration of neutropenia, faster recovery of fever, and shorter empiric antibiotic use, the administration of the colony-stimulating factors did not improve overall mortality and infection, including fungal-related mortality. In a phase IV randomized clinical trial comparing the effect of prophylactic treatment of 206 allogenic HSCT recipients with G-CSF, GM-CSF, or both G-CSF + GM-CSF, Wan et al. (2015) found IFD-related mortality after 600 days to be lower in the GM-CSF, G-CSF + GM-CSF groups as compared to G-CSF-alone group; 1.47, 1.45, and 11.59%, respectively (*P* = 0.016). The results of this trial may point in the direction of further studies with prophylactic GM-CSF, with or without G-CSF, to reduce IFD-related mortality in patients. Regarding IFD incidence, Hovi et al. (2000) and Wan et al. (2015) found no difference when G-CSF or GM-CSF were used with antifungals prophylactically in allogenic or autologous HSCT patients.

On an individual patient level, GM-CSF has shown promising results. Chen et al. (2017) reported success where GM-CSF was used as adjunct therapy to treat *Aspergillus* ventriculitis. GM-CSF was given in conjunction with voriconazole, amphotericin B, and caspofungin. After 2 years of therapy, the patient fully recovered and remained in remission. The role of GM-CSF in this case, was deemed significant by the authors, as fungal ventriculitis has a high mortality of 67% with conventional treatment. In a case of *Scedosporium apiospermum* with sub-optimal response to voriconazole, success was reported after switching to the antifungal micafungin co-administered with GM-CSF (Goldman et al., 2016).

The data above supports consideration for the use of G-CSF and GM-CSF, which constitutes our most familiar practice of immunotherapy, in particular G-CSF at the bedside. Concerns regarding thromboembolic events with colony-stimulating factor use are not well-founded based on findings of the Cochrane Review (Mhaskar et al., 2014). Perceived gains, however, are secondary until there can be firmer primary outcome data, such as reduction in infection or IFD-attributable mortality.

### Macrophage Colony-Stimulating Factor (M-CSF)

Macrophage colony-stimulating factor (also known as CSF-1), unlike G-CSF and GM-CSF, is not approved by the FDA for use in patients. M-CSF, as its name suggests, mainly promotes the growth of macrophages (reviewed in Hume and MacDonald, 2012). Kandalla et al. (2016) treated transplant-mouse models with M-CSF and found improved survival of the mice when challenged with *Aspergillus fumigatus*, from 10% in controls to 60% in M-CSF treated mice. Correspondingly, fungal burden in the lung, liver, and heart was lower in the M-CSF treated mice. Kandalla et al. (2016) found that M-CSF induced myeloid commitment of HSCs but not G-CSF. In the case series described by Nemunaitis et al. (1993) (reviewed in Hume and MacDonald, 2012), 46 stem cell transplantation patients with IFD were given recombinant human M-CSF with conventional antifungal treatment; there was better survival in the patients who received M-CSF, as compared to historical controls (27% vs. 5%, *P* = 0.027). There has been no follow up to these reported findings. In cancer, macrophages constitute both the tumor and tumor microenvironment, existing as tumor-associated macrophages, and can represent up to 50% of the tumor cell mass (reviewed in Medina-Echeverz et al., 2014). In this respect, the administration of M-CSF raises concerns of its theoretical predisposition to accelerate disease progression in cancer patients by enhancing this macrophage population. Hence, M-CSF, though it sounds like a cousin of the G-CSF and GM-CSF, and a potential therapeutic agent, might not be appropriate for the use upfront in cancer patients against fungal infections.

### Interferon-Gamma (IFN-γ)

Recombinant IFN-γ, also known as rIFN-γ1b, was approved by the US FDA in 2000 for the use in prophylaxis against infections for patients with chronic granulomatous disease, and to delay the progression of severe osteopetrosis (FDA, 2000). With greater understanding of the action of IFN-γ in fungal infections, some groups which have trialed IFN-γ as adjunctive therapy for patients with severe IFD have had encouraging results: in two patients with chronic pulmonary aspergillosis (Kelleher et al., 2006), in an HIV positive and two HIV-negative patients with pulmonary aspergillosis, when used in conjunction with GM-CSF (Bandera et al., 2008), seven kidney transplant patients with IFD (Armstrong-James et al., 2010), two cases of cerebral aspergillosis (Estrada et al., 2012; Mezidi et al., 2014), and a pediatric patient with disseminated candidiasis (Buddingh et al., 2015). All these case reports described recovery of the patients. Taking into account the likely positive publication bias, further clinical trials are vital to ascertain its efficacy.

There have been a few clinical trials for the use of IFN-γ in IFD. Pappas et al. (2004) conducted a phase 2, double-blind, placebo-controlled trial in HIV positive patients with acute *Cryptococcus* meningitis. The study showed a trend toward a more rapid sterilization of CSF in the patients who received IFN-γ compared to the placebo (*P* = 0.072). Their study also showed that the lower dosage of 100 μg of IFN-γ was sufficient to achieve similar results compared to the 200 μg recipients. IFN-γ was also well tolerated among the patients, with few significant adverse events. Another open label RCT compared the addition of IFN-γ to standard amphotericin B therapy in HIV positive patients with *Cryptococcus* meningitis (Jarvis et al., 2012). The study found that short-course IFNγ therapy significantly increased the rate of CSF *Cryptococcus* clearance (*P* = 0.02), with no significant increase in adverse events. In addition, they found that two doses of IFN-γ were as effective as six doses. Taken together, these two trials demonstrated that two doses of 100 μg of IFN-γ was sufficient for the faster clearance of fungal infection. Beyond *Cryptococcosis*, Delsing et al. (2014) conducted a clinical series between 2010 and 2013, using IFN-γ with standard antifungal therapy, the trial had to be terminated early due to low enrollment. The study recruited eight patients with invasive *Candida* and/or *Aspergillus* infections. The results were encouraging in that five of the eight patients treated with IFN-γ recovered from the IFD. Two of the ICU patients included in the study died due to infectious complications. One of the eight patients was lost to follow up. The authors observed that IFN-γ elevated IL-17 and IL-22 production. Monocyte and lymphocyte numbers also significantly increased after the initiation of IFN-γ therapy.

Numerous studies have produced promising results in the use of IFN-γ treatment, and encouraging results from the phase II studies discussed above, point to IFN-γ as a potential adjunct to standard treatment. Further larger clinical trials have to be conducted to establish the therapy’s efficacy and conditions for use.

### Cytokine Agonist Therapy

The capacity of the host immunity to recognize and respond to the fungal pathogen is mediated by a range of pathogen recognition receptors including Toll-like receptors (TLRs), such as TLRs 2, 4, 7, 9, and C-type lectin receptors (CLRs), e.g., Dectin-1. Accounts of the signaling pathways triggered upon recognition of the fungal ligand and the consequent host inflammatory responses elicited to date have been well-described by many esteemed publications (Gow et al., 2011; Romani, 2011; Wüthrich et al., 2012). All these constitute the host innate and adaptive immune arms against infection. Naturally, modulation and manipulation of these signaling pathways alter the net immune response and may alter the outcome of infection.

A modality considered was the use of an antigen or adjuvant to augment the inflammatory response. Various fungal-originated moieties may be utilized as antigens to activate the host Th1, Treg or Th17 cytokine arms, perceived to confer protection against infection. Examples of fungal antigens include Pep1p, a secreted fungal aspartic protease, and the various glycosylphosphatidylinositol (GPI)-anchored proteins of the fungal cell wall, as demonstrated in mice model studies (Bozza et al., 2009). The administration of cytosine guanine oligodeoxynucleotide (CpG, a TLR9 agonist) and the *Aspergillus* antigen Asp f16, induced Th1 priming in dendritic cells (Bozza et al., 2002). Furthermore, adoptive transfer of Asp f16-peptide specific cytotoxic T lymphocytes improved survival of *Aspergillus*-infected mice (Sun et al., 2012). While attempts continue to develop fungal-specific vaccines, immunocompromised individuals at risk of fungal infection may have limited capacity to respond to vaccination and the solution may lie in the elucidation of more potent adjuvants to elicit some protective immunity.

Imiquimod, a TLR-7 agonist, is a topical treatment used in actinic keratosis, external genital warts, and superficial basal cell carcinoma. In a novel adaption of such immune augmenting therapy, Erbagci et al. (2005) reported success with the use of topical imiquimod in an immunocompetent healthy patient who had lesions on her face for over 20 years caused by *Acremonium strictum*. The lesions been treated unsuccessfully with topical and systemic antifungals and cryotherapy. Finally the lesions were healed after changing to topical imiquimod and oral itraconazole. de Sousa et al. (2014) also reported favorable outcomes with their use of topical imiquimod in four patients with Chromoblastomycosis, a chronic subcutaneous lesion, most commonly caused by *Fonsecaea pedrosoi*. Marked improvement of the lesions was reported in all the patients with or without concurrent conventional antifungal medications.

Pentraxin-3 is a soluble pattern recognition receptor and acute phase protein. Pentraxin-3 has been implicated in host innate resistance against *Aspergillus* infections (Garlanda et al., 2002; Bozza et al., 2014). In a cohort of patients undergoing allogenic stem cell transplantation, genetic deficiency of PTX3 leading to defective PTX3 expression was associated with increased risk of invasive aspergillosis (IA) (Cunha et al., 2014). Hypothesizing from a rat model of IA in which treatment with PTX3 reportedly improved survival and reduced lung fungal burden (Lo Giudice et al., 2010), the administration of pentraxin-3 to complement conventional therapy may be a consideration.

## Cellular Therapy

### Granulocyte Transfusion (GTX)

Granulocyte transfusion involves transfusion of granulocytes, including neutrophils, from a donor to the patient. Neutrophils are required for killing fungal pathogens (Levitz and Diamond, 1985) and neutropenia is a well-established risk factor for IFD (Marr et al., 2002).

In theory, GTX restores the neutrophil count and augments the host’s defenses against fungi. However, the actual working of GTX was fraught with many problems which made it fall out of favor. Problems included low granulocyte counts, low quality and short lifespan of the granulocytes, compounded by the advent of newer antifungal drugs – details of which have been excellently reviewed in West et al. (2017). From the 1990s, the use of recombinant cytokines such as G-CSF, and better apheresis methods, have allowed larger doses of granulocytes to be delivered to the patient and has revived interest in this method (reviewed in Ravikumar et al., 2015; West et al., 2017). Price et al. (2015) observed that doses greater than 0.6 × 10^9^, ideally 1 × 10^9^ granulocytes per kilogram, seemed to have beneficial outcomes. In contrast, Valentini et al. (2017) compared the variable dosages given between the RCTs and concluded that dosage of the granulocytes did not translate to any clinical benefit to the patients.

West et al. (2017) summarized 97 cases, reporting that GTX together with G-CSF yielded an overall response rate of 50–90% in IFD. The authors also described seven IFD case series using G-CSF mobilized GTX. Six reported favorable results, while the 7th (Raad et al., 2013) reported therapeutic GTX treated patients with IA had a higher risk of not responding to antifungal treatment and had higher mortality compared to controls. West et al. (2017) also described instances of bacterial and fungal infections in adult and pediatric patients receiving GTX, with a very wide range of response rates, from 0% (Grigg et al., 1996) to 100% (Sachs et al., 2006), which makes it hard to make any conclusions.

In a Cochrane review of 11 GTX RCTs, Estcourt et al. (2015) found low grade evidence that prophylactic GTX may decrease the risk of bacterial or fungal infections. In a separate review by the same authors, they reviewed the use of therapeutic instead of prophylactic GTX (Estcourt et al., 2016). They compared 10 trials conducted from 1975 to 2015 and concluded conservatively that there is low grade evidence of GTX having any significant decrease in mortality rate. The statistical power of the 10 trials was insufficient to determine whether GTXs affected all-cause mortality. Moreover, only two trials (Seidel et al., 2008; Price et al., 2015) have been conducted within the past 15 years since G-CSF has been incorporated into routine practice to increase the granulocyte count, making the statistical power even weaker. While these two studies did not find statistical significance, both were also riddled with obstacles such as low enrolment rates and procedural obstacles, such as differences in granulocyte dosage. Disappointingly, an ongoing study of GTX, the transfusion of granulocytes for patients with FN, the GRANITE study (German Clinical Trials Register number DRKS00000218 and EudraCT number 2009-010700-28), was last updated “recruiting withdrawn before recruiting started” (Drks, 2017). RCTs of a potentially life-saving therapy are not only a scientific problem, but an ethical problem. It is very hard for clinicians and patients alike, knowing that there is a potential life-saving therapy, to withhold the therapy from the control group. It poses an ethical dilemma, on one hand, the individual patient is severely ill; on the other hand, the wider medical community needs rigorous scientific evidence.

### Adoptive T-Cell Therapy

Adoptive T-cell therapy involves the harvesting of T-lymphocytes from a patient or donor’s blood, stimulating the cells to grow and expand in an *in vitro* system, and these cells are subsequently re-infused back into the patient, primed for action.

Adoptive T-cell therapy is already in use against viral infections in HSCT patients (reviewed in Papadopoulou et al., 2016), but it is not in use yet to treat fungal infections, partly because of the technical challenges associated with the handling, manipulation and stimulation of cells. Firstly, we face technical problems of scaling up enrichment of the T-cells in accordance with good manufacturing practice (GMP) procedures for clinical use. Many groups have come up with ingenious strategies with the aim to target multiple pathogens in a shorter period of time (Tramsen et al., 2008; Khanna et al., 2011; Tramsen et al., 2013; Stuehler et al., 2015). Bacher et al. (2015) reported success on a method to prepare and isolate GMP-compliant T-helper cells reactive to *Aspergillus fumigatus* for adoptive transfer. The protocol and *Aspergillus fumigatus* lysate will be used in the ongoing clinical trial (Prüfplancode: 01-13IDWUE, EudraCT Nr. 2013-002914-11, German Clinical Trials Register number DRKS00007890) to assess their *in vivo* safety and efficacy. Next, the modality of immunosuppression also matters. For example, immunosuppressants such as cyclosporine A, commonly used in solid organ and stem cell transplantation compromises optimal T-cell response (Tramsen et al., 2014). Adoptive T-cell therapy might be most effective in transplantation settings where anti-GVHD prophylaxis is not required, such as T-cell depleted HSCT (reviewed in Papadopoulou et al., 2016).

As of now, there has only been one clinical trial for adoptive T-cell therapy in the context of patients with IFD. Perruccio et al. (2005) showed that 9 out of 10 transplant patients with IA had resolution of *Aspergillus* infection after receiving a single dose of *Aspergillus*-specific T-cell clones, compared to a response rate of only 53% (7 out of 13) in the control group. All nine subjects who had a resolution of infection also had normalization of galactomannan antigenemia levels within 6 weeks, while the controls had higher levels (*P* < 0.002 vs. controls). Another clinical trial (NCT02843321), reported by Sutrave et al. (2017), used a mix of donor-derived, infection specific T-cells against *Aspergillus*, and viruses such as AdV, influenza, EBV, CMV, VZV, and BKV. The study was completed at the end of 2017 and its results had not been published at the time of writing this paper. To date, the promise of adoptive T-cell therapy is best epitomized by the success recently reported by Tzannou et al. (2017) using third party-derived virus-specific T-cells against BKV, CMV, EBV, AdV, and HHV-6 in HSCT patients. We eagerly await the results of more such clinical trials in the pipeline for adoptive T-cell therapy to become a reality for patients with IFD.

### Chimeric Antigen Receptor (CAR) T-Cell Therapy

In this therapy, T-cells are modified with a disarmed genetically engineered virus to express a CAR. The CAR modification allows T-cells to execute their killing command without the need to bind to other receptors. In 2017, two CAR T-cell therapies were approved by the U.S. FDA for use in cancer, Kymriah (tisagenlecleucel) for B-cell acute lymphoblastic leukemia patients (FDA, 2017b) and Yescarta (axicabtagene ciloleucel) for B-cell lymphoma (FDA, 2017a). The approval of these CAR T-cell therapies was a historic action, making it the first gene therapy available in the United States.

There has only been one CAR T-cell construct adapting the fungal receptor Dectin-1 for *Aspergillus* to activate T-cells via chimeric CD28 and CD3-ζ (Kumaresan et al., 2014). There are concerns on the potential limitations of CAR T-cell therapy which may hamper its use in IFD. Side effects observed in patients undergoing the two approved CAR T-cell therapies included cytokine release syndrome and neurological toxicities (Neelapu et al., 2017; Schuster et al., 2017). Thus, we hope that technological progress will help surmount these difficulties to make CAR T-cell therapy for IFD a reality in the future.

### Natural Killer (NK) Cell Therapy

Natural killer cell therapy, also known as adoptive transfer of NK cells, is the transfer of NK cells from a donor to a patient. The NK cells, unlike T-cells, do not need to be antigen-primed as they are activated whenever they do not receive the inhibitory self-MHC I receptor binding (reviewed in Dahlberg et al., 2015).

*In vitro* human NK cells were demonstrated to have activity against a wide spectrum of fungi – *Aspergillus fumigatus* (Park et al., 2009; Bouzani et al., 2011; Schneider et al., 2016), *C. albicans* (Voigt et al., 2014), *Rhizopus oryzae* (Schmidt et al., 2013), *Paracoccidioides brasiliensis* (Jimenez and Murphy, 1984), *Cryptococcus neoformans* (Levitz et al., 1994), and various clinical isolates of the mucormycetes (Schmidt et al., 2016).

Currently, NK cell therapy is in its trial stage for cancers. Pre-emptive NK cell therapy has been trialed in a phase II clinical trial in haploidentical HSCT patients to prevent graft failure and to add antitumor effects with little risk of GVHD (Stern et al., 2012). The results seem to provide no apparent benefit or serious adverse events to patients at the dosage used. In the context of IFD, there has been no clinical trial to date using NK cells to treat IFD. Thus, NK cell therapy shows potential as an immunotherapeutic option against IFD, though much more work has to be done to prove its efficacy in human trials.

## Monoclonal Antibodies (mAbs)

Therapeutic mAbs progression into clinical trials are lacking for IFD, currently most of the mAbs for fungi are developed for diagnostic purposes. At the time of writing this paper, only two therapeutic mAbs for fungi have made it past phase I trials, 18B7 and Efungumab.

18B7 is a murine-derived antibody against the polysaccharide capsule of *C. neoformans* (Casadevall et al., 1998). In a phase I trial it was well tolerated in an HIV-positive subjects (Larsen et al., 2005). 18B7 mAb was subsequently modified with a radioisotope for radio-immunotherapy, which were effective in prolonging survival and reducing *C. neoformans* burden in mice, while sparing damage to epithelial or macrophage-like cells (Bryan et al., 2013). 18B7 behaved as a catalytic antibody that catalyzes hydrolysis of the *C. neoformans* polysaccharide capsule (Bowen et al., 2017). Other than the academic study of 18B7, the progress of 18B7 to phase II/III clinical trials has been on hold due to a lack of industrial support (Ostrosky-Zeichner et al., 2010).

Mycograb, also known as Efungumab, had made it through a multinational phase II clinical trial. Its modality of action was to target cell wall-associated fungal heat shock protein 90 and to synergize the antifungal activity of amphotericin B (Matthews et al., 2003). It had been tested to work together with standard antifungal therapy and showed a good overall response rate of 84% (Mycograb) vs. 49% (controls), reducing overall *Candida*-related mortality from 18% (controls) to 4% (Mycograb) (Pachl et al., 2006). However, the production of Mycograb was fraught with manufacturing quality control issues, and it was refused market authorization by the European Medicines Agency (EMA, 2007). Mycograb’s purported capacity to augment amphotericin B was later thought to be non-specific (Richie et al., 2012). An alternative C28Y variant was produced, but disappointingly did not demonstrate the same *in vitro* or *in vivo* efficiency as Mycograb (Louie et al., 2011; Richie et al., 2012). Another new potential mAb awaiting further development is a humanized mAb, mAbP6E7, against the cutaneous mycotic pathogen *Sporothrix* (de Almeida et al., 2017). We hope that in due time, we will see more support and initiatives in the field of therapeutic antibody development.

## Immunomodulation by Other Substances

Most pathogens induce cytokine production as part of the host immune response, which normally is a beneficial response to recruit leukocytes and eliminate the pathogen. In some situations, infections induce an excessively large inflammation, or a cytokine storm. Uncontrolled, this can sometimes cause more damage to the host than the pathogen itself, especially when the patient is suffering from other comorbidities. As such, immunomodulatory agents to attenuate the overactive immune response may benefit the patient (Chai et al., 2011).

### Vitamins

Vitamin D, also known as 25-hydroxyvitamin D, is produced by our bodies upon exposure to sunlight. Beyond its effects on bone, vitamin D also modulates the immune system. Vitamin D generally skews the immune response toward an anti-inflammatory profile. Interestingly, *Candida*-infected mice receiving low doses of vitamin D were reported to have significantly decreased fungal burden in their kidneys, compared with either control mice which did not receive vitamin D, or mice which received high doses of vitamin D (Lim et al., 2015). These observations were accounted for through low dose vitamin D-mediated suppression of SOCS3 and induction of vitamin D receptor binding to the vitamin D-response elements in the promoter of the gene encoding INF-γ. This highlighted the bimodal influence of vitamin D in host response against *Candida* and that extreme ranges of levels of vitamin D had adverse effects on the immunity, and *Candida* resistance. Studies are being done to determine if this dose-dependent effect of vitamin D supplementation for IFD can be translated into humans. In a phase I, open label study (NCT01222273), Nguyen et al. (2015) gave 6 months of daily vitamin D3 (cholecalciferol) supplementation to cystic fibrosis patients who had low levels of vitamin D and allergic bronchopulmonary aspergillosis. The study found *Aspergillus* induced IL-13 and IgE levels to be significantly decreased after 24 weeks (*P* = 0.04). Vitamin D supplementation as a therapy is an easily available, economical, and attractive intervention. Taking into account all these studies, it seems that cautious vitamin D supplementation may improve outcomes in fungal-related diseases.

Vitamin A, best known for its role in good eyesight, has been known to also have immunomodulatory effects. atRA is a metabolite of vitamin A, added to monocytes, challenged with UV-treated *C. albicans*, has been found to produce lower levels of pro-inflammatory cytokines TNFα, IL-6, and IL-12b, when compared to monocytes without atRA (Klassert et al., 2014). Klassert et al. (2017) studied the gene expression of monocytes incubated with atRA or vitamin D, and challenged with *Aspergillus fumigatus* and *C. albicans*. They found generally both atRA and vitamin D downregulated most genes related to cytokine and chemokine activity, and also regulated non-coding RNAs involved in immunity (Riege et al., 2017). A review of 6 cystic fibrosis patients with chronic necrotizing pulmonary aspergillosis, who received voriconazole and vitamin A supplementation, reported that all the patients survived and recovered, though a higher incidence of visual disturbances were reported (Cheng et al., 2010). The use of vitamin A or atRA has not been tested in animal models of fungal infection, and more studies have to be conducted to understand the feasibility of vitamin A as an adjunct immune modulator against the fungi.

### Repurposing of Existing Drugs

Drug development is a long, costly and arduous process, with only a handful of drug candidates eventually making it to the bedside. Repurposing of existing drugs has the benefit of shortcutting the process, while saving precious time and money. The drugs are already recognized, optimized in terms of pharmacodynamics, pharmacokinetics, safety and toxicity profiles, and have gone through the necessary regulatory processes. Some drugs that have been used for various diseases have been repurposed as adjuncts for the use in fungal infections. We would like to mention a few of these repurposed drugs that have made it to the clinical trial stage.

Deferasirox, is an iron chelator used for treatment of iron overload. In a mouse model, it was found to be efficacious against *Rhizopus*, which requires iron for growth (Ibrahim et al., 2007). A randomized, double-blinded, placebo-controlled trial repurposing deferasirox as an adjunct agent to amphotericin B therapy did not yield a positive result (DEFEAT Mucor study, NCT00419770; Spellberg et al., 2012). Unfortunately, statistical analysis showed that patients in the deferasirox arm of the trial were also more likely to have active malignancy, neutropenia and have received corticosteroid therapy, and patients given deferasirox had a higher mortality compared to the controls, which made interpretation of results inconclusive at best (Donnelly and Lahav, 2012; Spellberg et al., 2012). Optimistically, after the study results was published, Soman et al. (2012) reported seven patients who were prescribed deferasirox with standard treatment for mucormycosis where adequate surgical debridement was not feasible. The condition of these patients improved, and the authors attributed the success of the treatment to the patient group having minimal comorbidities. Thus, further RCTs are needed to establish the efficacy of this treatment.

Other drugs have had better results, such as the ASTRO-CM study (NCT01802385) which repurposed sertraline, a selective serotonin reuptake inhibitor antidepressant, as an adjunctive treatment for HIV-associated *Cryptococcus* meningitis (Rhein et al., 2016). Sertraline was found to have a growth-inhibitory effect, inhibiting the fungal protein synthesis (Zhai et al., 2012; Trevino-Rangel Rde et al., 2016). *In vivo*, sertraline reduced *Cryptococcus* burden in the mouse brain (*P* < 0.05), comparable to fluconazole (Zhai et al., 2012; Trevino-Rangel Rde et al., 2016). In the brain tissue, sertraline was concentrated 22 times higher than in the blood (Lewis et al., 2013), making it an ideal candidate for infection involving the central nervous system. Results of the trial were promising the rate of fungal clearance was better in the sertraline group, with suggestive decreased incidence of paradoxical IRIS and lower disease recurrence. The authors speculated that sertraline might also have immunoregulatory effects to explain the decreased IRIS and lower recurrence, but it requires more evidence from *in vitro* studies and RCTs.

Tamoxifen, an estrogen receptor antagonist, is currently in a phase II clinical trial to determine if it augments antifungal therapy against *Cryptococcus* meningitis (NCT03112031). Tamoxifen, like sertraline, crosses the blood–brain barrier and achieves high concentrations in the brain (Morello et al., 2003). Its role as an antifungal was identified, and observed to be synergistic with fluconazole (Wiseman et al., 1989; Dolan et al., 2009; Spitzer et al., 2011). In a murine model of *Cryptococcus* infection, the combination of tamoxifen and fluconazole reduced *Cryptococcus* brain burden significantly compared to untreated control (*P* = 0.001) (Butts et al., 2014). Tamoxifen worked by interfering with calcium homeostasis as an inhibitor of calmodulin (Beggs, 1993; Dolan et al., 2009; Butts et al., 2014). We hope to hear their results in future updates of the trial.

Other exciting drugs being considered include auranofin and ebselen, Auranofin is a gold complex originally used to treat rheumatoid arthritis. Ebelsen is an organoselenium drug with an anti-inflammatory predisposition. Both auranofin and ebelsen inhibit the thioredoxin reductase pathway (reviewed in May et al., 2018). Pivotal to this mode of action is that the thioreductase system, which is critical in maintaining cells in a reduced state, is different in humans (and hence are not affected by auranofin and ebelsen) in contrast to bacteria and fungi. Auranofin induces heme oxygenase 1 expression, which degrades heme and has anti-inflammatory properties (Kobayashi et al., 2006). Ebselen induces reactive oxygen species-mediated cytotoxicity in yeast (Azad et al., 2014). Both had been found to have *in vitro* activity against *Aspergillus* and *Candida* (Ngo et al., 2016; Wiederhold et al., 2017). It is to be highlighted though that the activity of auranofin and ebelsen are not specific to fungi, but also all bacteria, helminths and some protozoans (reviewed in May et al., 2018). Nonetheless should their efficacy be further validated, these drugs will have the potential to be studied in a range of antimicrobial human clinical trials.

## Promising Future Therapies

### Immunomodulation Through Probiotics and the Microbiome

To defeat pathogenic microorganisms, it used to be thought that we had to kill them with antimicrobials. There has been a paradigm shift in recent years – the concept of defeating microorganisms with microorganisms. The successful utilization of donor fecal transfer for recurrent *Clostridia* infection is one such example (van Nood et al., 2013). Probiotics, the beneficial microorganisms, work by an array of mechanisms, from directly competing with the fungi for nutrients and secreting inhibitory metabolites, to modulating cytokine levels (reviewed in Abedin-Do et al., 2015; Matsubara et al., 2016).

Studies with probiotics have generally yielded beneficial outcomes for patients. We focus on the probiotic yeast, *Saccharomyces boulardii*, which has been used to counter diarrhea. *S. boulardii* is able to modulate the immune system. It was found to stimulate secretory IgA production (Buts et al., 1990; Rodrigues et al., 2000; Qamar et al., 2001), and also modulates signaling pathways NF-κB and MAP kinase, ERK1/2, and p38 signaling – the details are excellently summarized in the review by Pothoulakis (2009). In a mouse model, *S. boulardii* was shown to work antagonistically to *Candida* species (Ducluzeau and Bensaada, 1982), inhibiting *Candida* gut translocation (Berg et al., 1993), inhibiting fungal hyphal and biofilm formation (Krasowska et al., 2009), and decreasing inflammation and *Candida* gut colonization (Jawhara and Poulain, 2007). A note of caution though, the administration of probiotics in neonates, severely debilitated, and immunocompromised patients is still subject to debate as there have been case reports of iatrogenic *S. boulardii* infections (reviewed in Goldenberg et al., 2015).

Clinical trial results are also optimistic for the use of *S. boulardii* as prophylaxis or adjunct therapy. A clinical trial compared the rates of fungal infections in very low birth weight infants with nystatin prophylaxis vs. oral *S. boulardii*; it found lower rates of *Candida* colonization and less feeding intolerance in those infants administered with *S. boulardii* (Demirel et al., 2013). A meta-analysis of 22 clinical trials found that pediatric patients with diarrhea had better outcomes when given *S. boulardii* probiotic supplementation (Feizizadeh et al., 2014). From the large body of evidence on the efficacy of *S. boulardii* supplementation, we believe it will be a useful adjunct therapy for fungal infection that is easily available and economically feasible for the patient. The advantages of probiotic supplementation too, are that patients see it as a “natural” alternative to drugs and may be more willing to consume it.

Our microbiota has been coined as an organ, forgotten and now rediscovered (O’Hara and Shanahan, 2006). The term microbiome is used to refer to all the microorganisms in our body, but usually refers to the bacterial aspect of the microbiome, as it is most researched. The mycobiome in particular, is termed to refer to the more neglected fungal aspect of the microbiome (reviewed in Sam et al., 2017). Both the micro- and myco-biomes play a significant role in modulating the immune system, also known as gut-immune axis. The gut microbiota can produce many metabolites that modulate the immune system, such as vitamins, short chain fatty acids, medium-chain fatty acids, secondary bile acids, bacteriocins, and antimicrobial peptides (reviewed in Sam et al., 2017).

The components of the mycobiome matter in disease outcomes. Chemically induced colitis in dectin-1 deficient mice was observed to be worse than in control mice (Iliev et al., 2012). On analysis of the dectin-1 deficient mice’s mycobiome, they found *Candida tropicalis*, an opportunistic pathogen, constituted a large portion of the mycobiome. Furthermore, fungal invasion of the colonic wall explained the poor outcome of these dectin-1 deficient mice. Upon supplementation of the dectin-1 deficient mice with either *S. fibuligera*, a non-pathogenic fungus, or with *C. tropicalis*, mice with *S. fibuligera* supplementation had much less weight loss than the mice that had *C. tropicalis* supplementation. It was also noted that there was less production of IL-17 and IFN-γ in *S. fibuligera* supplemented mice compared to *C. tropicalis* supplemented mice. Although *S. fibuligera* is not a probiotic, the study showed the importance of the microbial flora and how it could alter the outcome of colitis in mice.

Current therapy for patients undergoing immunosuppression frequently involves broad spectrum antibiotics and antifungal prophylaxis to reduce the risks of infection (reviewed in Maertens et al., 2012). The extent of their impact on both the microbiome and mycobiome, however, is not known – highlighting the collateral effects of broad spectrum antimicrobials on the commensal microbial flora which acts antagonistically against pathogens. More has to be done to determine the immunological impact of micro- and myco-biome interactions in humans, which will pave the way for future therapies to modulate the immune system through the micro- and mycobiota.

A summary of the clinical studies in this paper can be found in **Table 1**.

**Table 1:** Summary of human clinical studies involving immunomodulatory therapies.

Reference	Study type/clinical phase	Study cohort/Patients	Intervention	Treatment outcome/therapeutic response
**Cytokine therapy**
G-CSF, GM-CSF
Clark et al. (2005)	Meta-analysis of 13 clinical trials	Total of 1,518 patients in the 13 trials	Six studies used G-CSF, six used GM-CSF, one used G-CSF, and GM-CSF treatment	G-CSF and/or GM-CSF use reduced the duration of neutropenia andthe length of stay during febrileneutropenia episodes; however, it wasinconclusive whether administrationimproved infection-relatedmortality
Mhaskar et al. (2014)	Meta-analysis of 14 RCTs	1,553 patients with chemotherapy-induced febrile neutropenia in 14 trials	Treatment with antibiotics plus G-CSF or GM-CSF vs. antibiotics alone	Shorter duration of neutropenia, faster recovery of fever, shorter empiric antibiotic use, administration of the CSFs did not improve overall mortality and infection (including fungal)-related mortality
Wan et al. (2015)	Phase IV	206 allo-HSCT patients	Prophylactic treatment with G-CSF, GM-CSF, or G-CSF + GM-CSF	IFD-related mortality was lower in GM-CSF, G-CSF + GM-CSF group
M-CSF
Nemunaitis et al. (1993)	Phase I/II trials	Long term follow up of 46 stem cell transplantation patients	The experimental group was given recombinant human M-CSF daily with conventional antifungal treatment.	There was better survival in the patients who received M-CSF, compared against historical controls (27% vs. 5%)
Interferon-gamma
Pappas et al. (2004)	Phase II, double-blind, placebo-controlled trial	70 HIV positive patients with acute *Cryptococcus* meningitis (23 controls, 25 receiving 100 μg of IFN-γ, 22 receiving 200 μg of IFN-γ)	Patients received 100 or 200 μg of IFN-γ or placebo three times a week for 10 weeks, in addition to standard therapy consisting of intravenous amphotericin B, with or without flucytosine, for 14 days, followed by fluconazole.	Patients who received IFN-γ showed a trend toward rapid sterilization of cerebrospinal fluid (CSF). Lower dosage of 100 μg IFN-γ achieved similar results as the 200 μg IFN-γ recipients
Jarvis et al. (2012)	Open label RCT	88 HIV-infected patients infected with *Cryptococcus* meningitis (31 controls, 29 receiving 2 doses, and 20 receiving 6 doses)	Patients were randomized to receive standard antifungal therapy for 2 weeks (amphotericin B plus flucytosine), or standard antifungal therapy plus two doses of 100 μg IFN-γ, or standard antifungal therapy plus six doses of 100 μg IFNγ	The IFNγ therapies caused significantly faster CSF *Cryptococcus* clearance, with no significant increase in adverse events. Two doses of IFN-γ yielded similar outcomes as six doses
Delsing et al. (2014)	Open label clinical case series	11 patients with invasive *Candida* and/or *Aspergillus* infections, consisting of 3 placebo controls, 3 with IFN-γ, 5 more treated with IFN-γ included as therapy of last resort	The experimental group was administered IFN-γ, 50 μg/m^2^ body surface, three times a week, in addition to standard antifungal therapy	Five of eight patients treated with IFN-γ recovered from the IFD. Two of the patients admitted into the study as last resort passed away due to infectious complications. One of eight was lost to follow up. All three patients in the control group recovered
Cytokine agonist therapy
Erbagci et al. (2005)	Case report	One patient with recalcitrant hyalohyphomycosis caused by *Acremonium strictum*	Imiquimod topical 5% cream applied to area of fungal lesions and oral itraconazole antifungal treatment	Full regression of the fungal lesions after 2 months of therapy, and complete regression of all clinical and hematological abnormalities
de Sousa et al. (2014)	Case series	Four patients with Chromoblastomycosis, caused by *Fonsecaea pedrosoi*	Imiquimod topical 5% cream applied to area of fungal lesion, with or without antifungal treatment	After application of imiquimod cream, two patients had complete clearance of infection. The other two patientssaw improvement after usingimiquimod
**Cellular therapy**
Granulocyte transfusion (GTX)
West et al. (2017)	Review of GTX from case series, prophylactic GTX in eight RCTs, therapeutic GTX in five RCTs	Adult and pediatric patients with underlying malignancy and IFD and/or bacterial infection	The experimental groups were given prophylactic GTX or therapeutic GTX	Low-grade evidence that prophylactic GTX may reduce the incidence of fungaemia, but non-selective prophylaxis for all neutropenic patients does not prevent mortality due to IFD
Estcourt et al. (2015, 2016)	Meta-analysis of 11 RCTs and 10 RCTs, respectively	653 participants in total (Estcourt et al., 2015); 587 participants in total (Estcourt et al., 2016)	Estcourt et al. (2015) – The experimental groups were given prophylactic GTX vs. controls without GTX.Estcourt et al. (2016) – The experimental group were given therapeutic GTX vs. controls without GTX	Both Estcourt et al. (2015, 2016) found that prophylactic and therapeutic GTX, respectively, had low grade evidence for decreasing the risk of bacterial or fungal infections, or decreasing mortality rate. There was insufficient evidence to detect significant differences in mortality due to infection between GTX-treated patients and controls, and the GTX efficacy may be dose-dependent. The statistical power of the 10 trials is insufficient to determine whether GTXs affected all-cause mortality
Raad et al. (2013)	Case series, retrospective review	Patients with hematological malignancies, prolonged neutropenia, and proven or probable invasive aspergillosis (IA)	Retrospective review of outcomes with or without therapeutic GTX	Therapeutic GTX treated patients with IA had a higher risk of not responding to antifungal treatment and had higher mortality compared to controls
Seidel et al. (2008)	Phase III randomized controlled trial	74 patients with neutropenia were randomized to receive standard antimicrobial/antifungal therapy plus GTX (*n* = 38) vs. the no-GTX control arm (*n* = 34)	Therapeutic GTX was administered to patients, in addition to standard antimicrobial/antifungal therapy	No statistical significance between the two groups
Price et al. (2015), The RING study	Phase III, open label, multicenter randomized controlled trial	Patients with neutropenia from 14 centers were randomized to receive standard antimicrobial therapy plus GTX (*n* = 56), or the control arm without GTX (*n* = 58)	Patients received daily GTX until neutrophil count recovery, resolution or improvement of infection, toxicity or 42 days, whichever was earlier. G-CSF was used to increase the granulocyte count in donors	No statistical significance was found between the GTX arm and the control arm, challenges encountered included low enrolment and variation in granulocyte dosage. Subjects who received >0.6 billion granulocytes/kg tended to have better outcomes than those receiving a lower dose of granulocytes
Adoptive T-cell therapy
Perruccio et al. (2005)	Phase I/II clinical trial	Transplant patients with evidence of IA (*n* = 10), controls (*n* = 13)	T-cells from donors were stimulated with *Aspergillus* antigen, selected for non-recipient reactive clones, and expanded. Patients were randomized to receive treatment with liposomal amphotericin B alone, with or without, single infusion of donor pathogen-specific T-cells	Nine out of 10 patients had resolution of *Aspergillus* infection after receiving a single dose of *Aspergillus*-specific T-cell clones, compared to a response rate of only 53% (7 out of 13) in the control group
Tzannou et al. (2017)	Phase II clinical trial	38 patients with 45 viral infections, consisting of 31 with a single virus infection and seven with two virus infections	Virus-specific T-cell lines for five viruses, Epstein–Barr virus (EBV), adenovirus (AdV), cytomegalovirus (CMV), BK virus (BKV), and human herpesvirus 6 (HHV-6) were manufactured from donors. All patients had at least a single infusion of partially	The patients had cumulative complete or partial response rate of 92%. Of special mention is by week 6, 13 of 14 patients with BKV-associated hemorrhagic cystitis had complete resolution of gross hematuria
			HLA-matched virus-specific T-cells, 11 had a second infusion after 4 weeks, and four patients had three infusions	
**Monoclonal antibodies**
Larsen et al. (2005)	Phase I clinical trial	20 HIV-positive patients who had a history of culture positive *Crypotcoccus* meningitis, with resolution of symptoms at least 12 weeks prior to the study	Patients were divided into six groups of escalating doses of 18B7 therapeutic monoclonal antibody against *C. neoformans*. The changes in serum cryptococcal antigen titres were monitored	18B7 antibodies was generally well tolerated at lower doses. At higher concentrations above 1 mg/kg, subjects developed infusion-associated back and muscle pain. They established that 18B7 can be safely given to subjects in doses below 1 mg/kg
Pachl et al. (2006)	Multinational phase III clinical trial	139 patients who had culture-positive *Candida* infection	The experimental group received intravenous Mycograb every 12 h for 5 days plus lipid-amphotericin B, while controls received lipid-amphotericin B alone	The complete overall response by day 10 was 84% for the Mycograb group and 48% for controls, and other indicators were also better for the Mycograb group, such as, better clinical response rate, *Candida*-attributable mortality, and rate of culture-confirmed clearance of the infection
**Immunomodulation by other substances**
Vitamins
Nguyen et al. (2015)	Phase I, open label clinical trial	Cystic fibrosis patients who had low levels of vitamin D and allergic bronchopulmonary aspergillosis	The experimental group was given 6 months of daily vitamin D3 (cholecalciferol) supplementation	*Aspergillus* induced IL-13 and IgE levels to be significantly decreased after 24 weeks (*P*=0.04)
Repurposing of existing drugs
Spellberg et al. (2012), DEFEAT Mucor study	Phase II, randomized, double-blinded, placebo-controlled trial	20 patients with proven or probable mucormycosis, 11 with deferasirox treatment, and nine placebo	Patients were randomized to receive treatment with liposomal amphotericin B plus/minus deferasirox (20 mg/kg/day for 14 days)	Patients given deferasirox had a higher mortality compared to the controls. The patients in the deferasirox arm of the trial were more likely to have active malignant cancer, neutropenia and corticosteroid therapy, and less likely to receive concurrent antifungals, which makes interpretation of results inconclusive
Rhein et al. (2016), ASTRO-CM study	Open label, dose-finding study	172 HIV-infected adult patients with *Cryptococcus* meningitis	The experimental group received adjunctive sertraline at doses of 100–400 mg/day, in addition to standard antifungal therapy	The rate of fungal clearance was better in the sertraline group, led to lower recurrence, and possibly decreased incidence of paradoxical immune reconstitution inflammatorysyndrome
**Promising future therapies**
Immunomodulation through probiotics and the microbiome
Demirel et al. (2013)	Randomized, comparative trial	Very low birth weight, preterm infants with gestational age ≤1,500 g	The infants were either given oral *Saccharomyces boulardii* prophylaxis (five billion colony forming unit per day) added to breast milk or formula once a day, starting with the first feed until discharged, or oral nystatin suspension every 8 h	In the *S. boulardii* group, there was lower rates of sepsis, *Candida* colonization and less feeding intolerance. *S. boulardii* was not found to grow in any of the positive blood cultures. Both groups observed no serious effects from the *S. boulardii* and nystatin
Feizizadeh et al. (2014)	Meta-analysis review of 22 randomized controlled trials	Pediatric patients with diarrhea	The experimental group was given *S. boulardii*	*S. boulardii* supplementation resulted in better outcomes, such as shorter duration of diarrhea, and reduced stool frequency. It did not cause any serious adverse events


## Concluding Remarks – Are We Closer?

**FIGURE 1:**
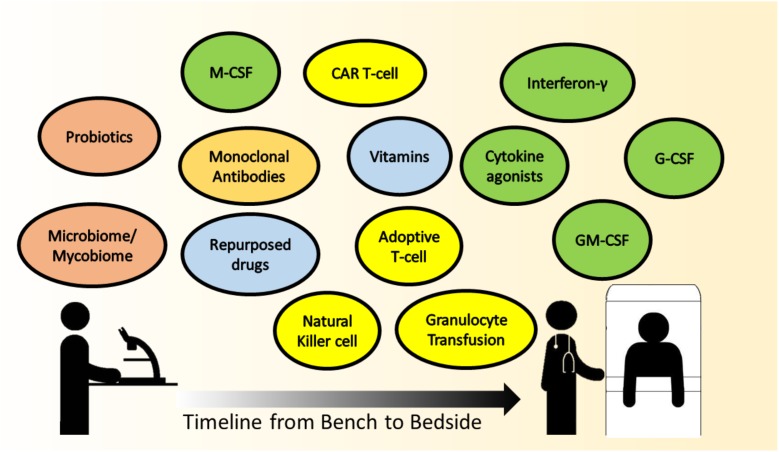
The timeline approximating the translation of the various immune-modulating strategies for IFD, from scientific research (bench) to clinical implementation (bedside). *Colored circles* represent the therapy headings mentioned in this paper, *green* – cytokine therapy, *yellow* – cellular therapy, *orange* – monoclonal antibodies, *blue* – other immunomodulatory agents, and *peach* – promising future therapies,. Therapies that are in scientific research and have the potential to be translated are on the *left*, with a continuum gradient of translation toward therapies that are closer to bedside use toward the *right*.

With the advancements in medical sciences, there is a trend toward a combination of treatment strategies to optimize management of complicated medical conditions. As has been reviewed here, immunomodulatory options for IFD are abound, but which strategies are most likely to be translatable to the bedside in the near future? Colony stimulating factors, mainly G-CSF and to a lesser extent GM-CSF, are being administered in chemotherapy-induced FN to reduce duration of neutropenia (Smith et al., 2006). Recombinant human IFN-γ is not a standard of care in IFD, but is selectively given with conventional antifungals in patients with difficult-to-treat IFD. The practice of GTX in IFD remains controversial. should be highlighted that indications for GTX, if so instituted, ought to be as a temporary measure to a more definitive end-point, such as the perceived recovery of neutrophil count. Patients with chronic diseases, and those requiring repeated hospitalizations are at risk of low vitamin D levels (Lim et al., 2015). Some units do actively screen and replenish vitamin D in their patients as per international guidelines, not specifically against IFD, but rather for bone health (Aloia, 2011). Adoptive T-cell transfer and CAR T-cell therapies are extremely novel and have the potential to be game-changers in treatment, perhaps in the foreseeable future. Adoptive T-cell infusion is already being administered against viral infections post-stem cell transplant, such as CMV (Blyth et al., 2013; Tzannou et al., 2017). The manipulation of the micro- and mycobiome, as state-of-art and intellectually stimulating, remains at this point an academic venture for understanding dysbiosis and disease pathogenesis. The timeline approximating the translation of the various immune modulating strategies for IFD to clinical implementation at the bedside is depicted in **Figure 1**.

It is imperative to highlight at this juncture, that in facing IFD, conventional antifungal drugs remains the backbone of any treatment. The current state of immunotherapy, at this stage, augments conventional treatment as an adjunct to optimize response and outcomes. Developing novel immunomodulatory strategies is at the forefront of medicine today. Considering the revolution of immunotherapies in treating cancer, immunomodulatory treatment options of IFD may ride on the wave of progress in cancer immunotherapy in the coming years.

Immunotherapy and antifungal therapy are our two-pronged strategy to win against fungi. Just like a battle in an electronic game, immunotherapy is our potion to strengthen ourselves, while antifungal therapy is our potion to weaken the enemy. In an electronic battle though, when you run out of potions, the gravest consequence is your avatar faints, you lose the battle, and you revive it after the match. In real life, patients’ lives are at stake in the battle against IFD. We face a constant battle against IFD, and we hope there will be a greater awareness of the need for more research and clinical trials in the exciting realm of IFD.

## Author Contributions

QS and LC wrote the review paper, together with WY, CS, and MC. All authors contributed to manuscript revision, read, and approved the submitted version.

## Conflict of Interest Statement

The authors declare that the research was conducted in the absence of any commercial or financial relationships that could be construed as a potential conflict of interest.

## Abbreviations

AdV: adenovirus
AML: acute myelogenous leukemia
atRA: all-trans retinoic acid
BKV: BK virus
CAR: chimeric antigen receptor
CMV: cytomegalovirus
CSF: cerebrospinal fluid
EBV: Epstein–Barr virus
FDA: U.S. Food and Drug Administration
FN: febrile neutropenia
G-CSF: granulocyte colony-stimulating factor
GM-CSF: granulocyte-macrophage colony-stimulating factor
GTX: granulocyte transfusion
GVHD: graft vs. host disease
HHV-6: human herpesvirus-6
HSCT: hematopoietic stem cell transplant
IFD: invasive fungal disease
IFN-γ: interferon-gamma
IRIS: immune reconstitution inflammatory syndrome
mAbs: monoclonal antibodies
M-CSF: macrophage colony-stimulating factor
MHC: major histocompatibility complex
NK cell: natural killer cell
RCTs: randomized controlled trials
SOCS3: suppressor of cytokine signaling 3
VZV: Varicella-Zoster virus
